# *N*′-[(*E*)-4-Meth­oxy­benzyl­idene]-2-(5-meth­oxy-2-methyl-1*H*-indol-3-yl)acetohydrazide

**DOI:** 10.1107/S1600536813027050

**Published:** 2013-10-19

**Authors:** Mehmet Akkurt, Joel T. Mague, Shaaban K. Mohamed, Antar A. Abelhamid, Mustafa R. Albayati

**Affiliations:** a Department of Physics, Faculty of Sciences, Erciyes University, 38039 Kayseri, Turkey; b Department of Chemistry, Tulane University, New Orleans, LA 70118, USA; c Chemistry and Environmental Division, Manchester Metropolitan University, Manchester M1 5GD, England; d Chemistry Department, Faculty of Science, Mini University, 61519 El-Minia, Egypt; e Department of Chemistry, Faculty of Science, Sohag University, 82524 Sohag, Egypt; f Kirkuk University, College of Science, Department of Chemistry, Kirkuk, Iraq

## Abstract

The conformation adopted by the title compound, C_20_H_21_N_3_O_3_, in the crystal is ‘J’-shaped and appears to be at least partially directed by a weak intra­molecular C—H⋯N hydrogen bond. In the crystal, mol­ecules are linked by N—H⋯O hydrogen bonds, forming dimers with *R*_2_^2^(8) motifs. Furthermore, these dimers connect to each other *via* C—H⋯O and N—H⋯O hydrogen bonds to form a three-dimensional network.

## Related literature

For general medical applications of non-steriodal anti-inflammatory drugs (NSAIDs), see: Richy *et al.* (2004 ▶). For the undesirable side effects of such drugs, see: Allison *et al.* (1992 ▶); McMahon (2001 ▶); Rocha *et al.* (2001 ▶); Halen *et al.* (2009 ▶). For a similar structure, see: Mague *et al.* (2013 ▶). For hydrogen-bond motifs, see: Bernstein *et al.* (1995 ▶).
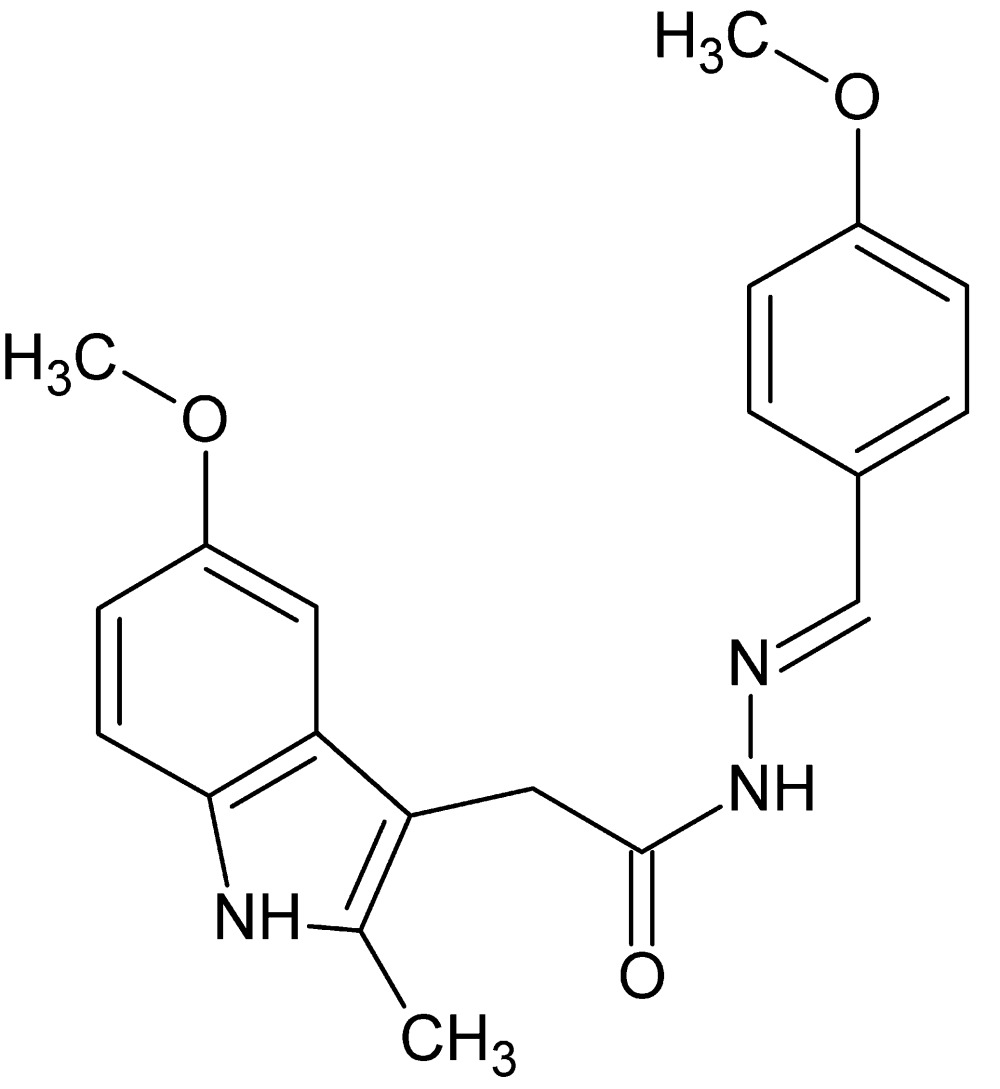


## Experimental

### 

#### Crystal data

C_20_H_21_N_3_O_3_
*M* *_r_* = 351.40
Triclinic, 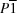

*a* = 7.1894 (2) Å
*b* = 10.4055 (3) Å
*c* = 12.4403 (4) Å
α = 107.983 (2)°
β = 92.451 (2)°
γ = 97.882 (2)°
*V* = 873.24 (5) Å^3^
*Z* = 2
Cu *K*α radiation
μ = 0.74 mm^−1^
*T* = 100 K
0.14 × 0.12 × 0.08 mm

#### Data collection

Bruker D8 VENTURE PHOTON 100 CMOS diffractometer
Absorption correction: multi-scan (*SADABS*; Bruker, 2013 ▶) *T*_min_ = 0.85, *T*_max_ = 0.94
8928 measured reflections
3121 independent reflections
2579 reflections with *I* > 2σ(*I*)
*R*_int_ = 0.029

#### Refinement

*R*[*F* ^2^ > 2σ(*F* ^2^)] = 0.036
*wR*(*F* ^2^) = 0.090
*S* = 1.04
3121 reflections
246 parameters
H atoms treated by a mixture of independent and constrained refinement
Δρ_max_ = 0.19 e Å^−3^
Δρ_min_ = −0.18 e Å^−3^

### 

Data collection: *APEX2* (Bruker, 2013 ▶); cell refinement: *SAINT* (Bruker, 2013 ▶); data reduction: *SAINT*; program(s) used to solve structure: *SHELXTL* (Sheldrick, 2008 ▶); program(s) used to refine structure: *SHELXL2013* (Sheldrick, 2008 ▶); molecular graphics: *ORTEP-3 for Windows* (Farrugia, 2012 ▶); software used to prepare material for publication: *WinGX* (Farrugia, 2012 ▶) and *PLATON* (Spek, 2009 ▶).

## Supplementary Material

Crystal structure: contains datablock(s) global, I. DOI: 10.1107/S1600536813027050/sj5357sup1.cif

Structure factors: contains datablock(s) I. DOI: 10.1107/S1600536813027050/sj5357Isup2.hkl

Additional supplementary materials:  crystallographic information; 3D view; checkCIF report

## Figures and Tables

**Table 1:** Hydrogen-bond geometry (Å, °)

*D*—H⋯*A*	*D*—H	H⋯*A*	*D*⋯*A*	*D*—H⋯*A*
N1—H1⋯O2^i^	0.88 (2)	2.04 (2)	2.9212 (17)	174.0 (18)
N2—H2⋯O2^ii^	0.920 (18)	1.988 (18)	2.9025 (16)	171.9 (15)
C4—H4⋯O3^iii^	0.95	2.50	3.410 (2)	161
C11—H11*B*⋯N3	0.99	2.36	2.8373 (19)	109
C20—H20*A*⋯O1^iv^	0.98	2.49	3.215 (2)	131

Symmetry codes: (i) 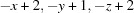; (ii) 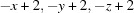; (iii) 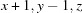; (iv) 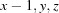.

**Fig. 1.**
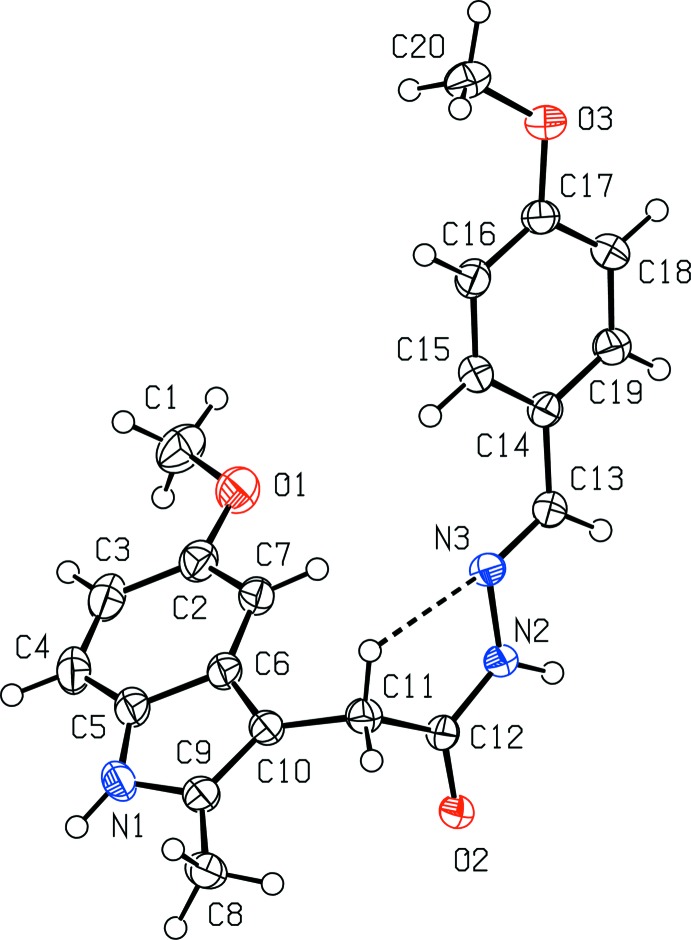
Perspective view of the title compound with 50% probability displacement ellipsoids.

**Fig. 2.**
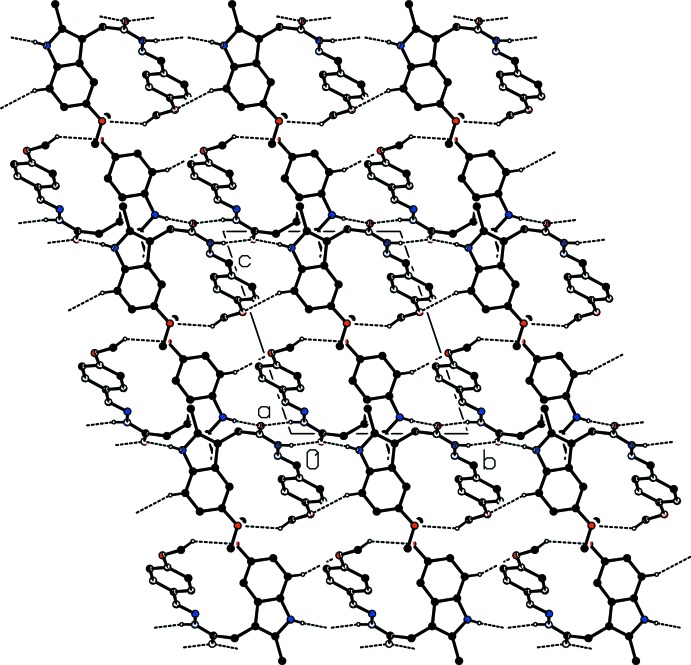
Packing viewed along a showing the hydrogen bonds as dotted lines.

## supplementary crystallographic information

### 1. Comment

Indomethacin like other non-steroidal anti-inflammatory drugs (NSAIDs) is widely used in treatment of pain, fever, and inflammation (Richy *et al.*, 2004). Prolonged administration of such drugs is commonly associated with several undesired side-effects. The most common of these are gastrointestinal hemorrhage, ulceration, and decreased renal function (Allison *et al.*, 1992; McMahon 2001; Rocha *et al.*, 2001). The existence of a free carboxylic acid group in the parent drug has been considred to be the major factor in establishing superficial stomach erosion, particularly in the corpus region of the stomach (Halen *et al.*, 2009). Thus, it was considered essential to mask or to remove this functional group in order to produce a safer and more tolerant prodrug profile. Following this reasoning, we report here the synthesis and crystal structure of the title compound.

The "J" shaped conformation of the title molecule (I) is shown in Fig. 1. The bond lengths and bond angles of (I) compare well with those in related compounds (Mague *et al.*, 2013).

In the crystal, the molecules form inversion dimers with *R*_2_^2^(8) motifs (Bernstein *et al.*, 1995) through N—H···O hydrogen bonds (Fig. 2, Table 1). In addition, the dimers are linked by C—H···O and N—H···O hydrogen bonds (Table 1), forming a three-dimensional network.

### 2. Experimental

A mixture of 233 mg (1 mmol) 2-(5-methoxy-2-methyl-1*H*-indol-3-yl)acetohydrazide and 136 mg (1 mmol) of 4-methoxybenzaldehyde in 30 ml ethanol containing few drops of glacial acetic acid was refluxed for 5 h. The reaction mixture was allowed to cool to room temperature and the excess solvent was evaporated under *vacuum*. The residual solid was collected, washed with cold ethanol and recrystallized from ethanol. Colourless blocks of X-ray quality were obtained. *M*.p. 453–455 K.

### 3. Refinement

The H atoms of the amino group were found in the difference Fourier maps, and were refined freely. C-bound H atoms were placed geometrically and refined using a riding model with C—H = 0.95 - 0.99 Å, and with *U*_iso_(H) = 1.2 or 1.5*U*_iso_(C).

### Crystal data

**Table:**

C_20_H_21_N_3_O_3_	*Z* = 2
*M**_r_* = 351.40	*F*(000) = 372
Triclinic, *P*1	*D*_x_ = 1.336 Mg m^−^^3^
Hall symbol: -P 1	Cu *K*α radiation, λ = 1.54178 Å
*a* = 7.1894 (2) Å	Cell parameters from 6305 reflections
*b* = 10.4055 (3) Å	θ = 3.8–68.2°
*c* = 12.4403 (4) Å	µ = 0.74 mm^−^^1^
α = 107.983 (2)°	*T* = 100 K
β = 92.451 (2)°	Block, colourless
γ = 97.882 (2)°	0.14 × 0.12 × 0.08 mm
*V* = 873.24 (5) Å^3^	

### Data collection

**Table:**

Bruker D8 VENTURE PHOTON 100 CMOS diffractometer	3121 independent reflections
Radiation source: INCOATEC IµS micro–focus source	2579 reflections with *I* > 2σ(*I*)
Mirror monochromator	*R*_int_ = 0.029
Detector resolution: 10.4167 pixels mm^-1^	θ_max_ = 68.2°, θ_min_ = 3.8°
ω scans	*h* = −8→8
Absorption correction: multi-scan (*SADABS*; Bruker, 2013)	*k* = −11→12
*T*_min_ = 0.85, *T*_max_ = 0.94	*l* = −14→12
8928 measured reflections	

### Refinement

**Table:**

Refinement on *F*^2^	0 restraints
Least-squares matrix: full	H atoms treated by a mixture of independent and constrained refinement
*R*[*F*^2^ > 2σ(*F*^2^)] = 0.036	*w* = 1/[Σ^2^(*F*_o_^2^) + (0.0398*P*)^2^ + 0.2493*P*] where *P* = (*F*_o_^2^ + 2*F*_c_^2^)/3
*wR*(*F*^2^) = 0.090	(Δ/σ)_max_ = 0.001
*S* = 1.04	Δρ_max_ = 0.19 e Å^−^^3^
3121 reflections	Δρ_min_ = −0.18 e Å^−^^3^
246 parameters	

### Special details

**Table:**

Geometry. Bond distances, angles *etc*. have been calculated using the rounded fractional coordinates. All su's are estimated from the variances of the (full) variance-covariance matrix. The cell e.s.d.'s are taken into account in the estimation of distances, angles and torsion angles
Refinement. Refinement on *F*^2^ for ALL reflections except those flagged by the user for potential systematic errors. Weighted *R*-factors *wR* and all goodnesses of fit *S* are based on *F*^2^, conventional *R*-factors *R* are based on *F*, with *F* set to zero for negative *F*^2^. The observed criterion of *F*^2^ > σ(*F*^2^) is used only for calculating -*R*-factor-obs *etc*. and is not relevant to the choice of reflections for refinement. *R*-factors based on *F*^2^ are statistically about twice as large as those based on *F*, and *R*-factors based on ALL data will be even larger.

### Fractional atomic coordinates and isotropic or equivalent isotropic displacement parameters (Å^2^)

**Table:**

	*x*	*y*	*z*	*U*_iso_*/*U*_eq_	
O1	0.71418 (17)	0.52381 (11)	0.54515 (9)	0.0430 (4)	
O2	0.98707 (13)	0.84392 (9)	1.03898 (8)	0.0292 (3)	
O3	−0.13304 (14)	0.95693 (11)	0.60125 (9)	0.0371 (3)	
N1	0.79353 (17)	0.35490 (13)	0.91900 (12)	0.0351 (4)	
N2	0.76381 (16)	0.90476 (12)	0.94351 (10)	0.0260 (3)	
N3	0.58416 (16)	0.88308 (12)	0.89050 (9)	0.0265 (3)	
C1	0.7349 (3)	0.44162 (19)	0.43283 (14)	0.0480 (6)	
C2	0.7324 (2)	0.46796 (15)	0.63133 (13)	0.0345 (5)	
C3	0.7787 (2)	0.33672 (16)	0.61512 (15)	0.0388 (5)	
C4	0.7991 (2)	0.28921 (15)	0.70661 (15)	0.0384 (5)	
C5	0.7752 (2)	0.37351 (15)	0.81367 (14)	0.0326 (4)	
C6	0.72832 (19)	0.50583 (14)	0.83158 (13)	0.0296 (4)	
C7	0.7039 (2)	0.55117 (15)	0.73782 (13)	0.0310 (4)	
C8	0.7797 (2)	0.48079 (17)	1.12353 (14)	0.0379 (5)	
C9	0.76673 (19)	0.47320 (15)	1.00178 (14)	0.0317 (5)	
C10	0.72383 (19)	0.56677 (14)	0.95159 (12)	0.0281 (4)	
C11	0.67910 (19)	0.70663 (14)	1.01121 (12)	0.0284 (4)	
C12	0.82053 (19)	0.82164 (14)	0.99829 (11)	0.0256 (4)	
C13	0.5515 (2)	0.97217 (14)	0.84341 (11)	0.0272 (4)	
C14	0.3702 (2)	0.96285 (14)	0.78169 (11)	0.0269 (4)	
C15	0.2179 (2)	0.86146 (14)	0.77499 (12)	0.0290 (4)	
C16	0.0484 (2)	0.85462 (15)	0.71417 (12)	0.0302 (4)	
C17	0.0283 (2)	0.95182 (15)	0.66076 (12)	0.0296 (4)	
C18	0.1787 (2)	1.05399 (15)	0.66754 (12)	0.0322 (5)	
C19	0.3475 (2)	1.05862 (15)	0.72627 (12)	0.0305 (5)	
C20	−0.2801 (2)	0.84238 (17)	0.57471 (14)	0.0380 (5)	
H1	0.855 (3)	0.295 (2)	0.9360 (16)	0.052 (5)*	
H1A	0.86220	0.41770	0.42890	0.0720*	
H1B	0.71490	0.49270	0.38010	0.0720*	
H1C	0.64190	0.35780	0.41200	0.0720*	
H2	0.847 (2)	0.9799 (18)	0.9426 (14)	0.037 (4)*	
H3	0.79600	0.28050	0.54110	0.0470*	
H4	0.82900	0.20030	0.69610	0.0460*	
H7	0.66820	0.63830	0.74730	0.0370*	
H8A	0.66310	0.43210	1.13970	0.0570*	
H8B	0.79780	0.57680	1.17140	0.0570*	
H8C	0.88670	0.43840	1.13960	0.0570*	
H11A	0.67420	0.71790	1.09300	0.0340*	
H11B	0.55260	0.71370	0.98090	0.0340*	
H13	0.64810	1.04650	0.84880	0.0330*	
H15	0.23020	0.79590	0.81260	0.0350*	
H16	−0.05350	0.78380	0.70910	0.0360*	
H18	0.16500	1.12090	0.63150	0.0390*	
H19	0.45010	1.12800	0.72920	0.0370*	
H20A	−0.23210	0.75960	0.53170	0.0570*	
H20B	−0.38450	0.85740	0.52910	0.0570*	
H20C	−0.32500	0.83130	0.64510	0.0570*	

### Atomic displacement parameters (Å^2^)

**Table:**

	*U*^11^	*U*^22^	*U*^33^	*U*^12^	*U*^13^	*U*^23^
O1	0.0543 (7)	0.0399 (6)	0.0312 (6)	0.0116 (5)	0.0040 (5)	0.0044 (5)
O2	0.0263 (5)	0.0249 (5)	0.0361 (5)	0.0024 (4)	−0.0050 (4)	0.0112 (4)
O3	0.0322 (6)	0.0354 (6)	0.0412 (6)	0.0052 (5)	−0.0101 (5)	0.0103 (5)
N1	0.0263 (7)	0.0250 (7)	0.0565 (9)	0.0034 (5)	−0.0021 (6)	0.0177 (6)
N2	0.0243 (6)	0.0238 (6)	0.0290 (6)	0.0014 (5)	−0.0035 (5)	0.0091 (5)
N3	0.0257 (6)	0.0267 (6)	0.0251 (6)	0.0044 (5)	−0.0018 (5)	0.0060 (5)
C1	0.0488 (11)	0.0495 (10)	0.0348 (9)	0.0063 (8)	0.0034 (8)	−0.0016 (8)
C2	0.0298 (8)	0.0309 (8)	0.0389 (8)	0.0030 (6)	0.0015 (6)	0.0063 (7)
C3	0.0322 (8)	0.0288 (8)	0.0469 (9)	0.0022 (6)	0.0044 (7)	0.0007 (7)
C4	0.0269 (8)	0.0232 (8)	0.0604 (11)	0.0041 (6)	0.0038 (7)	0.0064 (7)
C5	0.0217 (7)	0.0253 (7)	0.0494 (9)	0.0010 (6)	−0.0006 (6)	0.0117 (7)
C6	0.0201 (7)	0.0245 (7)	0.0421 (8)	0.0007 (5)	−0.0014 (6)	0.0093 (6)
C7	0.0271 (8)	0.0257 (7)	0.0383 (8)	0.0038 (6)	−0.0007 (6)	0.0079 (6)
C8	0.0301 (8)	0.0363 (9)	0.0516 (10)	−0.0003 (6)	−0.0071 (7)	0.0240 (8)
C9	0.0199 (7)	0.0287 (8)	0.0469 (9)	−0.0017 (6)	−0.0035 (6)	0.0159 (7)
C10	0.0212 (7)	0.0250 (7)	0.0388 (8)	0.0006 (5)	−0.0021 (6)	0.0131 (6)
C11	0.0266 (7)	0.0280 (8)	0.0312 (7)	0.0028 (6)	−0.0002 (6)	0.0113 (6)
C12	0.0275 (8)	0.0227 (7)	0.0246 (7)	0.0053 (6)	0.0004 (6)	0.0044 (6)
C13	0.0298 (8)	0.0240 (7)	0.0266 (7)	0.0031 (6)	0.0004 (6)	0.0072 (6)
C14	0.0297 (8)	0.0257 (7)	0.0237 (7)	0.0063 (6)	0.0006 (6)	0.0049 (6)
C15	0.0323 (8)	0.0250 (7)	0.0301 (7)	0.0063 (6)	0.0008 (6)	0.0089 (6)
C16	0.0284 (8)	0.0257 (7)	0.0336 (8)	0.0026 (6)	0.0015 (6)	0.0060 (6)
C17	0.0296 (8)	0.0303 (8)	0.0264 (7)	0.0094 (6)	−0.0022 (6)	0.0042 (6)
C18	0.0362 (8)	0.0313 (8)	0.0316 (8)	0.0066 (6)	−0.0019 (6)	0.0136 (7)
C19	0.0298 (8)	0.0301 (8)	0.0316 (8)	0.0026 (6)	−0.0004 (6)	0.0111 (6)
C20	0.0293 (8)	0.0443 (9)	0.0367 (8)	0.0029 (7)	−0.0040 (6)	0.0098 (7)

### Geometric parameters (Å, º)

**Table:**

O1—C1	1.422 (2)	C14—C15	1.393 (2)
O1—C2	1.3776 (19)	C15—C16	1.388 (2)
O2—C12	1.2422 (17)	C16—C17	1.390 (2)
O3—C17	1.3653 (18)	C17—C18	1.389 (2)
O3—C20	1.425 (2)	C18—C19	1.377 (2)
N1—C5	1.386 (2)	C1—H1A	0.9800
N1—C9	1.383 (2)	C1—H1B	0.9800
N2—N3	1.3818 (16)	C1—H1C	0.9800
N2—C12	1.3475 (19)	C3—H3	0.9500
N3—C13	1.2810 (19)	C4—H4	0.9500
N1—H1	0.88 (2)	C7—H7	0.9500
N2—H2	0.920 (18)	C8—H8A	0.9800
C2—C3	1.406 (2)	C8—H8B	0.9800
C2—C7	1.382 (2)	C8—H8C	0.9800
C3—C4	1.384 (2)	C11—H11A	0.9900
C4—C5	1.383 (2)	C11—H11B	0.9900
C5—C6	1.415 (2)	C13—H13	0.9500
C6—C10	1.434 (2)	C15—H15	0.9500
C6—C7	1.400 (2)	C16—H16	0.9500
C8—C9	1.490 (2)	C18—H18	0.9500
C9—C10	1.369 (2)	C19—H19	0.9500
C10—C11	1.501 (2)	C20—H20A	0.9800
C11—C12	1.514 (2)	C20—H20B	0.9800
C13—C14	1.460 (2)	C20—H20C	0.9800
C14—C19	1.398 (2)		
			
C1—O1—C2	118.05 (13)	C14—C19—C18	121.03 (14)
C17—O3—C20	117.85 (13)	O1—C1—H1A	109.00
C5—N1—C9	109.09 (13)	O1—C1—H1B	109.00
N3—N2—C12	122.52 (12)	O1—C1—H1C	109.00
N2—N3—C13	114.94 (12)	H1A—C1—H1B	109.00
C9—N1—H1	120.9 (12)	H1A—C1—H1C	109.00
C5—N1—H1	126.0 (13)	H1B—C1—H1C	109.00
N3—N2—H2	118.7 (10)	C2—C3—H3	120.00
C12—N2—H2	118.8 (10)	C4—C3—H3	120.00
O1—C2—C3	123.67 (14)	C3—C4—H4	121.00
O1—C2—C7	115.23 (14)	C5—C4—H4	121.00
C3—C2—C7	121.10 (15)	C2—C7—H7	120.00
C2—C3—C4	120.20 (16)	C6—C7—H7	120.00
C3—C4—C5	118.84 (15)	C9—C8—H8A	109.00
C4—C5—C6	121.74 (15)	C9—C8—H8B	109.00
N1—C5—C6	107.17 (14)	C9—C8—H8C	110.00
N1—C5—C4	131.07 (15)	H8A—C8—H8B	109.00
C5—C6—C10	107.06 (13)	H8A—C8—H8C	109.00
C7—C6—C10	134.17 (14)	H8B—C8—H8C	109.00
C5—C6—C7	118.73 (14)	C10—C11—H11A	109.00
C2—C7—C6	119.35 (15)	C10—C11—H11B	109.00
N1—C9—C8	120.08 (14)	C12—C11—H11A	109.00
N1—C9—C10	109.40 (14)	C12—C11—H11B	109.00
C8—C9—C10	130.47 (15)	H11A—C11—H11B	108.00
C6—C10—C11	126.49 (13)	N3—C13—H13	119.00
C9—C10—C11	126.29 (13)	C14—C13—H13	119.00
C6—C10—C9	107.22 (13)	C14—C15—H15	119.00
C10—C11—C12	113.58 (12)	C16—C15—H15	119.00
N2—C12—C11	119.29 (12)	C15—C16—H16	120.00
O2—C12—N2	118.72 (13)	C17—C16—H16	120.00
O2—C12—C11	121.97 (13)	C17—C18—H18	120.00
N3—C13—C14	121.97 (13)	C19—C18—H18	120.00
C13—C14—C19	119.12 (13)	C14—C19—H19	119.00
C15—C14—C19	118.23 (13)	C18—C19—H19	119.00
C13—C14—C15	122.65 (13)	O3—C20—H20A	110.00
C14—C15—C16	121.06 (14)	O3—C20—H20B	109.00
C15—C16—C17	119.73 (14)	O3—C20—H20C	109.00
O3—C17—C16	124.68 (14)	H20A—C20—H20B	109.00
C16—C17—C18	119.73 (13)	H20A—C20—H20C	109.00
O3—C17—C18	115.59 (14)	H20B—C20—H20C	109.00
C17—C18—C19	120.21 (15)		
			
C1—O1—C2—C3	3.0 (2)	C10—C6—C7—C2	174.92 (15)
C1—O1—C2—C7	−177.73 (15)	C7—C6—C10—C9	−177.33 (16)
C20—O3—C17—C18	169.28 (13)	C5—C6—C7—C2	−2.2 (2)
C20—O3—C17—C16	−11.6 (2)	C5—C6—C10—C9	0.04 (16)
C9—N1—C5—C6	−2.29 (16)	C8—C9—C10—C11	0.6 (3)
C5—N1—C9—C10	2.37 (17)	N1—C9—C10—C6	−1.46 (16)
C9—N1—C5—C4	176.09 (16)	N1—C9—C10—C11	178.14 (13)
C5—N1—C9—C8	−179.76 (13)	C8—C9—C10—C6	−179.03 (15)
C12—N2—N3—C13	179.16 (13)	C6—C10—C11—C12	−64.10 (18)
N3—N2—C12—C11	−5.17 (19)	C9—C10—C11—C12	116.38 (16)
N3—N2—C12—O2	176.44 (12)	C10—C11—C12—O2	−65.23 (17)
N2—N3—C13—C14	178.67 (12)	C10—C11—C12—N2	116.43 (14)
O1—C2—C3—C4	178.35 (14)	N3—C13—C14—C19	−176.06 (13)
C7—C2—C3—C4	−0.9 (2)	N3—C13—C14—C15	3.6 (2)
C3—C2—C7—C6	2.4 (2)	C13—C14—C19—C18	−179.70 (13)
O1—C2—C7—C6	−176.95 (13)	C15—C14—C19—C18	0.7 (2)
C2—C3—C4—C5	−0.7 (2)	C13—C14—C15—C16	−179.08 (13)
C3—C4—C5—N1	−177.38 (15)	C19—C14—C15—C16	0.6 (2)
C3—C4—C5—C6	0.8 (2)	C14—C15—C16—C17	−1.3 (2)
N1—C5—C6—C7	179.22 (13)	C15—C16—C17—C18	0.8 (2)
C4—C5—C6—C7	0.7 (2)	C15—C16—C17—O3	−178.29 (13)
C4—C5—C6—C10	−177.19 (14)	O3—C17—C18—C19	179.57 (13)
N1—C5—C6—C10	1.37 (16)	C16—C17—C18—C19	0.4 (2)
C7—C6—C10—C11	3.1 (3)	C17—C18—C19—C14	−1.2 (2)
C5—C6—C10—C11	−179.56 (13)		

### Hydrogen-bond geometry (Å, º)

**Table:**

*D*—H···*A*	*D*—H	H···*A*	*D*···*A*	*D*—H···*A*
N1—H1···O2^i^	0.88 (2)	2.04 (2)	2.9212 (17)	174.0 (18)
N2—H2···O2^ii^	0.920 (18)	1.988 (18)	2.9025 (16)	171.9 (15)
C4—H4···O3^iii^	0.95	2.50	3.410 (2)	161
C11—H11*B*···N3	0.99	2.36	2.8373 (19)	109
C20—H20*A*···O1^iv^	0.98	2.49	3.215 (2)	131

Symmetry codes: (i) −*x*+2, −*y*+1, −*z*+2; (ii) −*x*+2, −*y*+2, −*z*+2; (iii) *x*+1, *y*−1, *z*; (iv) *x*−1, *y*, *z*.
